# Left diaphragmatic hernia following thoracoabdominal aortic repair: A case report

**DOI:** 10.1016/j.ijscr.2020.04.038

**Published:** 2020-05-11

**Authors:** Andrea Castillo Barbosa, Carlos J. Pérez Rivera, Luis Jaime Tellez, Paulo Cabrera Rivera, Alejandro González-Orozco, Manuel Mosquera Paz

**Affiliations:** a Department of General Surgery, Fundación Cardioinfantil – Instituto de Cardiología, Bogotá, Colombia; b Universidad de los Andes, Colombia; c Fundación Cardioinfantil – Instituto de Cardiología, Bogotá, Colombia

**Keywords:** MRI, magnetic resonance imaging, CT-scan, computed tomography scan, Diaphragmatic hernia, Laparoscopic approach, Thoracoabdominal aortic repair, Case report

## Abstract

**Introduction:**

Diaphragmatic hernias are somewhat rare complications of thoracoabdominal interventions. Given their late clinical manifestations and misdiagnosis, their incidence is unknown. These hernias have a high mortality risk when an emergency intervention is warranted due to complications from visceral strangulation.

**Case presentation:**

We present the case of a 67-year-old male with prior history of thoracoabdominal aortic repair, who reconsults due to upper gastrointestinal bleeding. Upon arrival, imaging shows a left diaphragmatic herniation with migration of the stomach, omentum and spleen to the thoracic cavity. Through laparoscopic approach, a left diaphragmatic hernial defect is identified with protrusion of half the stomach, omentum and the posterior aspect of the spleen with a sub capsular tear. Additionally, a severe adhesion syndrome on the chest wall and diaphragm were also evident, with entrapment of the inferior lobe of the left lung. The contents were successfully reduced, however pulmonary decortication and extensive adhesiolysis through thoracoscopy was required for complete extraction, enabling a primary repair without tension.

**Conclusions:**

We present an infrequent pathology without an established incidence, which has relevant clinical and surgical implications at any level of care, in this case requiring interdisciplinary management. The suspicion of diaphragmatic hernia in a patient with past medical history of thoracoabdominal aortic repair with non-specific gastrointestinal symptoms is essential. We emphasize the importance of clinical suspicion of this complication once the surgical precedent has been identified.

## Highlights

• Diaphragmatic hernias are unusual thoracoabdominal complications, often misdiagnosed given its late clinical manifestations.
• Diaphragmatic hernias requiring emergency intervention are high-risk due to visceral strangulation.
• Clinical suspicion must be considered in a gastrointestinal symptoms with prior thoracoabdominal aortic repair.
• Immediate surgical intervention may be considered as the gold standard in delayed diaphragmatic rupture.

## 1 Introduction

Diaphragmatic hernias are unusual complications of thoracoabdominal interventions [1], corresponding to the migration of abdominal viscera into the pleural cavity through a diaphragmatic defect secondary to a pressure gradient between the peritoneum and the chest cavity [2]. Diaphragmatic hernias can be classified into congenital and acquired, with blunt trauma the most common etiology in acquired causes (73–88 % of cases), followed by penetrating trauma (12–32 % of cases) [3]. Postoperative iatrogenic diaphragmatic hernias are very rare [4], the actual incidence being unknown given that in a significant number of patients a hernia can remain silent, manifesting symptoms years later and some are even incidental findings in other pathologies [5]. Diaphragmatic hernias have been described following laparoscopic hepatectomies, gastrectomies, esophagectomies, splenectomies and nephrectomies, among others [6,7]. Since visceral strangulation is an emergency, as well as a diagnostic dilemma [1], its prompt diagnosis and treatment are essential. We present the case of a 67-year-old male with prior history of thoracoabdominal aortic repair, who reconsults six months later to our institution with nonspecific gastrointestinal symptoms, where upon a left diaphragmatic herniation with migration was identified requiring joint management between the groups of Gastrointestinal Surgery and Thoracic Surgery to achieve its correction. This case report is written in accordance to surgical case report (SCARE) criteria [8].

## 2 Case presentation

A 67-year-old male with prior history of diaphragmatic trauma eight years earlier and subsequent diaphragmatic hernia approached through thoracotomy, requiring open thoracoabdominal aortic reconstruction in 2017 due to a suprarenal abdominal aortic aneurysm; reconsults six months later to our emergency department with coffee ground vomit, general paleness and moderate dehydration. At his arrival, non-variceal upper gastrointestinal bleeding was suspected, undergoing an esophagogastroduodenoscopy without evidence of active bleeding and a chest x-ray that identified an image of a lower left pulmonary consolidation. A chest CT-scan was performed, illustrating a left diaphragmatic hernia with protrusion of the gastric body into the ipsilateral hemithorax.

**Fig. 1:**
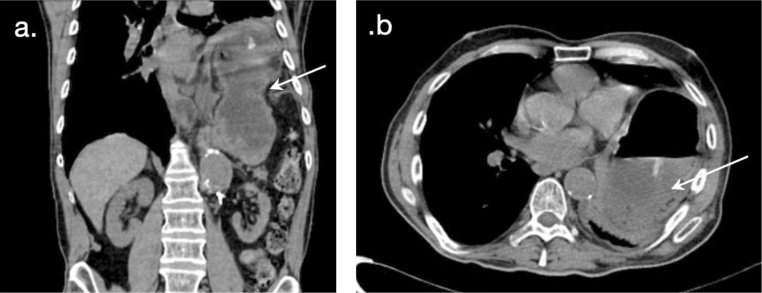
**A**–**B**. Pre-operative thoracoabdominal computed tomography scan: the seven-centimeter left hemi-diaphragmatic defect associated with herniation of the fundus, body and gastric antrum, along with the spleen.

Given the findings, an abdominal CT-scan was performed identifying a seven centimeter left hemi-diaphragmatic defect associated with herniation of the fundus, body and gastric antrum, along with the spleen (Fig. 1A and B). Due to the patient’s severe malnutrition, parenteral nutritional repletion was necessary since the passage of an advanced nutrition probe was not possible due to the anatomical alteration generated by the hernial defect.

**Fig. 2:**
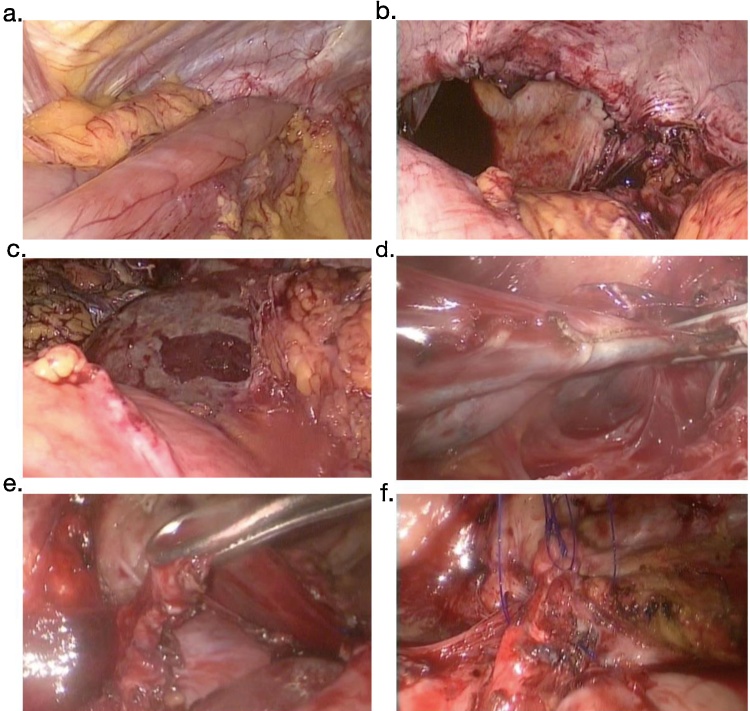
**A**–**F**. Intra-operative images: the left diaphragmatic hernial defect of seven by five centimeters with protrusion of half the stomach, omentum and the posterior aspect of the spleen with sub capsular tear, with preservation of the hiatus [A—C]. Once the Thoracic Surgery group had performed extensive adhesiolysis and decortication, the extraction of the protruding sac without damaging the spleen was attained, enabling a primary repair without tension [D—F].

Fifteen days after admission, the patient underwent abdominal laparoscopic surgery with evidence of a large left diaphragmatic hernia with protrusion of half the stomach, omentum and the posterior aspect of the spleen. Additional findings were the spleen had a sub capsular tear, as well as a severe adhesion syndrome on the chest wall and diaphragm with entrapment of the inferior lobe of the left lung. A hernial defect of seven by five centimeters was found, with preservation of the hiatus. The reduction of the herniated organs was achieved through abdominal laparoscopy, however a complete dissection was not possible due to the splenic interposition (Fig. 2A–C). The Thoracic Surgery group, through a thoracoscopic approach, performed extensive adhesiolysis and decortication, achieving the extraction of the protruding sac without damaging the spleen thus enabling a primary repair without tension (Fig. 2D–F).

**Fig. 3:**
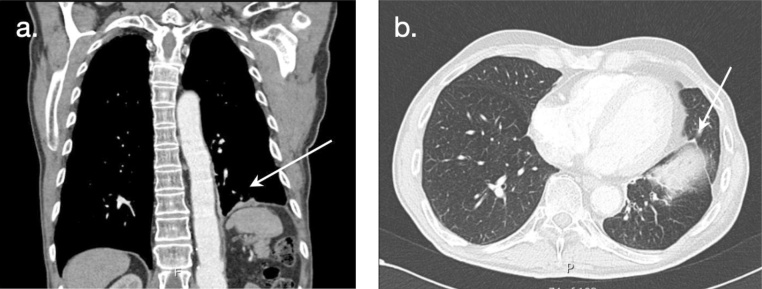
**A**–**B**. Post-operative thoracoabdominal computed tomography scan: a satisfactory correction of the diaphragmatic hernia.

Twenty-four hours postoperatively, the patient tolerated the liquid diet, presenting an uneventful recovery, being discharged on the fifth postoperative day with normal food intake. Six months later he was reviewed with an abdominal CT-scan showing a satisfactory diaphragmatic hernial correction (Fig. 3A-B).

## 3 Discussion

According to the literature, diaphragmatic hernias result in 10 % of diaphragmatic injuries, including blunt trauma, penetrating trauma and iatrogenic lesions [5]. Diaphragmatic hernias as complications of thoracoabdominal aortic repairs are rare. Its precise incidence is unknown, probably due to the lack of diagnosis given its late clinical manifestations, incidental finding or even high misdiagnosis rate [1,7]. In the absence of strangulated or obstructed viscera, diaphragmatic hernias may be diagnosed months or even years after the initial surgery [6]. In different series, a late presentation of a diaphragmatic hernia was reported in 5–62 % of the cases, with the longest delay being 35 years [4,9].

Grasping instruments, electrocautery and dissection forceps near the diaphragm may cause diaphragmatic injuries during surgery. Missed diaphragmatic tears present progressive elongation due to stress produced by the pressure gradient between the thorax and abdomen resulting in an intra-thoracic herniation [4,6]. Any defect is likely to increase in size as a result of the pressure gradient, which can reach up to 100 mmHg during Valsalva maneuvers such as coughing and physical activity [5]. This pathology may present with acute or chronic symptoms, or it can be evident as an incidental finding. The intensity of its clinical presentation will depend on the size and nature of the herniated organ [7]. The chronic clinical features most commonly associated are upper abdominal pain, lower chest pain, nausea, dyspnea, and gastroesophageal reflux after meals due to the gastric obstruction and reduction in lung volume; nevertheless, these may develop in an acute setting consisting of severe epigastric pain, vomiting, and intestinal obstruction [10,11]. Since there are no specific clinical symptoms, diaphragmatic hernias should be suspected in patients with past history of thoracoabdominal interventions. The operative mortality for the correction of the hernial defect is approximately 10 % [11], however the mortality rate is often associated with emergency complications such as strangulation or visceral perforation, anywhere from 20 to 80% [1,4]. A mortality rate of 30 % has been evidenced in cases of diaphragmatic hernia with bowel strangulation [12].

Diagnosis may be achieved through chest X-ray, MRI, CT-scan or abdominal ultrasonography; in some cases, invasive approaches as diagnostic laparoscopy and thoracoscopy may be considered. Chest radiography has been recognized as the most appropriate test for the diagnosis of diaphragmatic hernias. Some literature indicates that plain chest X-ray is diagnostic in 73 % of the cases [5], while others establish that even though it is a good screening method, it only detects abnormality in 50 % of the patients [13]. Computed tomography (CT-scan) of the abdomen can be classified as the best imaging method, with a high sensitivity ranging from 14 to 82% and a high specificity from 76 to 100% [5]; yet, according to the literature, the specificity is only 50 % for right diaphragmatic herniations [14]. Helical computed tomography can further improve the diagnosis with a sensitivity of 100 % for left diaphragmatic herniations, while those on the right side have a sensitivity of 76 % [2]. The tomographic signs that can reveal the presence of a diaphragmatic hernia are: a disruption of the diaphragmatic dome, especially if the tear is close to the costal insertion of diaphragm; the hourglass sign adjacent to the defect representing the constriction of the herniated organs; and a blurring of the diaphragm in extensive defects. In our patient’s case, the abnormality was not detected through the chest X-ray due to the interposition of a left basal pneumonic process, resulting in the CT-scan as the imaging modality that revealed the diaphragmatic herniation.

Surgery is the first line of treatment for this pathology, and some cases warranting emergency intervention in symptomatic hernias with visceral strangulation. According to the literature, immediate surgical intervention may be considered as the gold standard treatment of delayed diaphragmatic rupture [15]. As for asymptomatic diaphragmatic hernias, its correction can be differed depending on the patient’s condition [6]. In our patient’s case, having discarded visceral strangulation, nutritional replenishment was prioritized prior to performing the surgical procedure.

The laparoscopic approach is appropriate in clinically stable patients, good general condition, and especially those who with a left diaphragmatic hernia, resulting in a shorter length of stay [4]. Laparotomy is suggested in the acute event or in unstable patients in order to examine the abdominal organs, perform adhesiolysis and expose the visceral ischemic areas [6]. Thoracotomy is considered by some authors for thoracic defects in the absence of abdominal pathology; however, it has been shown that these patients have longer lengths of stay and a greater risk of prolonged mechanical ventilation postoperatively [6]. For small defects, primary repair with non-absorbable material by separate suture is indicated, meanwhile in wide defects or in weak muscles, the synthetic grafts can be used to avoid excessive tension [2,11]. In our patient’s case, the primary closure was completed with separate non-absorbable polypropylene suture via thoracoscopy without tension.

## 4 Conclusion

We present the case of an infrequent pathology without an established incidence, which has relevant clinical and surgical implications at any level of care. The suspicion of diaphragmatic hernia in a patient with past medical history of thoracoabdominal aortic repair with non-specific gastrointestinal symptoms is essential. The pertinent images according to the patient’s clinical condition should be done to promptly identify this pathology and to establish the most appropriate surgical approach.

## Declaration of Competing Interest

The authors declare they have no conflicts of interest.

## Funding

This research did not receive any specific grant from funding agencies in the public, commercial, or not-for-profit sectors.

## Ethical approval

The Ethical and Research Committee of the Fundación Cardioinfantil – IC and the General Surgery Research Group at the Fundación Cardioinfantil – IC.

## Consent

Written consent was obtained from the patient for publication of this report. Any details identifying the individuals to the clinical history and images associated were eliminated as to remain anonymous.

## Author contribution

Castillo Barbosa A, Perez Rivera CJ-, Orozco-Gonzalez A and Cabrera P designed the report, analyzed the data, and wrote the paper. Mosquera Paz M and Tellez LJ collected patient’s data and were the perioperative attending physicians. All authors read and approved the final manuscript.

## Registration of research studies

N/A.

## Guarantor

Perez Rivera Carlos Jose.

**Email:** cjperezrivera@gmail.com.

**Address**: Calle 163A N° 13B-60, Bogota, Cundinamarca, Colombia.

**Telephone:** + 57 (031) 667 2727 Ext. 12301.

## Provenance and peer review

Not commissioned, externally peer-reviewed.

## Funding

There was financing by Fundacion Cardioinfantil Bogota Colombia.
